# Emergence of Achromobacter xylosoxidans Bacteremia in a Tertiary Care Center: A Case Report and Literature Review

**DOI:** 10.7759/cureus.68084

**Published:** 2024-08-29

**Authors:** Karthiga Dhakshna Murthi, Swetha Naik, Shanmuga Leela Arumugam, Manonmoney J, Leela KV

**Affiliations:** 1 Microbiology, SRM Medical College Hospital and Research Centre, Kanchipuram, IND

**Keywords:** achromobacter xylosoxidans, nosocomial bacteria, incisional hernia repair, antimicrobial susceptibility pattern, bacteriemia

## Abstract

*Achromobacter xylosoxidans (A. xylosoxidans) *is an opportunistic pathogen that is responsible for various nosocomial and community-acquired infections. It is often found in patients with cystic fibrosis or chronic lung diseases.

Here, a 70-year-old female patient presented to an emergency department with complaints of diffuse abdominal pain and distension, on and off giddiness, and generalized body pain for one month with a known case of diabetes and hypertension. The patient had no history of nausea, vomiting, constipation/loose stools, or fever at the time of arrival. Then, the patient was admitted with a provisional diagnosis of incisional hernia. However, the patient developed a fever after she had undergone surgery for an incisional hernia. The blood culture reveals the growth of *A. xylosoxidans. *The patient responded well to treatment with intravenous antibiotics piperacillin/tazobactam and meropenem for five days. The literature on bacteremia caused by *A. xylosoxidans* in incisional hernia patients is reviewed in this study, along with the distinct antimicrobial susceptibility pattern.

## Introduction

*Achromobacter xylosoxidans (A. xylosoxidans*) is an opportunistic microorganism associated with many nosocomial epidemics [1]. It is a gram-negative bacillus, aerobic, motile organism. It is widely present in the environment and primarily responsible for healthcare-associated infections. In the limited reports available on this subject, *A. xylosoxidans* bacteremia has predominantly been observed in patients with malignant conditions, particularly those with hematological malignancies. One of the most extensive series included 52 cases of bacteremia diagnosed over 14 years in 46 cancer patients. Consistent with earlier studies, catheter-related infections were the primary source of bacteremia in the current cohort. *A. xylosoxidans* infections are typically considered healthcare-associated, a finding reinforced by our experience, where 92.3% of cases were acquired in the hospital [2]. Because of their biochemical similarities, *Achromobacter *spp. are often misidentified using conventional methods as both common (e.g., *Acinetobacter *spp., *Pseudomonas aeruginosa, Burkholderia cepacia complex, Stenotrophomonas maltophilia*) and uncommon (e.g.*, Ralstonia *spp. and *Pandoraea *spp.) non-fermenting gram-negative bacilli [2-5]. In some cases, it can be detrimental to a patient's health as it can spread illnesses to those with existing medical conditions [6]. Patients with intrusive devices and respiratory illnesses and immunocompromised individuals are susceptible to infections for* A. xylosoxidans. *There have been rare reports of *A. xylosoxidans*-related endocarditis, which is associated with a significant death rate [6,7].

Antimicrobial resistance mechanism

Apart from having multi-drug efflux systems, *A. xylosoxidans *also shows AmpC-type intrinsic β-lactamase and OXA-type β-lactamase resistance. It exhibits intrinsic resistance to a wide range of antibiotics, including cephalosporins, aztreonam, and ertapenem. Additionally, treating *A. xylosoxidans* infections can be challenging due to its ability to acquire genes that provide transferable resistance. The production of biofilms, which most strains naturally possess, further contributes to their virulence and resistance to antimicrobial agents [3,6,8,9] Therefore, it is crucial to evaluate the efficacy of novel antibiotic compounds against this *Pseudomonas*-like bacterial species. Timely diagnosis and effective treatment are essential for achieving a favorable outcome.

## Case presentation

A 70-year-old female patient with a known history of hypertension and diabetes mellitus was brought to the emergency department with complaints of generalized body pains, intermittent giddiness, and abdominal pain for the past one month. There was no history of fever at the time of admission.

The patient underwent a laparotomy 15 years ago for a uterine tumor and a meshplasty procedure for an incisional hernia 10 years ago. She attained menopause 20 years ago. She has normal sleep and appetite, as well as normal bowel and bladder habits.

On general examination, the patient was clinically stable, conscious, oriented, and afebrile. Systemic examination revealed unremarkable respiratory and cardiovascular findings. Neurological examination was normal with no focal abnormalities. Abdominal examination revealed normal bowel sounds and abnormalities of both supra- and infra-umbilical regions. On admission, vital signs were as follows: blood pressure 120/80 mmHg, heart rate 84 bpm, temperature 36.7 °C, respiratory rate 17 breaths per minute, and oxygen saturation 98% on ambient air. Routine blood investigations on admission showed mildly elevated white blood cells, erythrocyte sedimentation rate count, and poor glycemic control (Table 1).

**Table 1:** Routine blood investigations done on the day of admission and after the procedure RBC: red blood cell, WBC: white blood cell, ESR: erythrocyte sedimentation rate, RBS: random blood sugar, HbA1c: glycosylated hemoglobin, SGOT/AST: aspartate aminotransferase, SGPT/ALT: alanine aminotransferase

Investigation parameters	Results		Reference range
	On admission (27.8.2022)	After procedure (8.9.2022)	
Hemoglobin (g/dL)	11.4 g/dl	9.9 g/dl	Female: 12-15 g/dl
Total RBC	4.2 million/cu.mm	3.6 million/cu.mm	Female: 3.8-4.8 million/cu.mm
WBC count (cells/mm^3^)	11,390/cu.mm	16,810/cu.mm	4000-11000/cu.mm
Platelets	2,92,000/cu.mm	2,87,000/cu.mm	1,50,000-4,10,000/cu.mm
ESR	38 mm/hr	51 mm/hr	Female: 0-20 mm/hr
RBS	189 mg/dl	206 mg/dl	70-140 mg/dl
HbA1c	9.6	7.0	<5.6 non-diabetic; >6.5 risk of developing DM
Urea (blood)	17 mg/dl	21 mg/dl	17-43 mg/dl
Creatinine (serum)	0.8 mg/dl	0.6 mg/dl	Female: 0.6-1.2 mg/dl
Total bilirubin (serum)	0.39 mg/dl	0.19 mg/dl	0.5-1.0 mg/dl
Direct bilirubin (serum)	0.10 mg/dl	0.04 mg/dl	Upto 0.3 mg/dl
Indirect bilirubin (serum)	0.29	0.15	0.2-0.8 mg/dl
Sodium (serum)	138 mmol/L	138 mmol/L	136-145 mmol/L
Potassium (serum)	4.1 mmol/L	3.8 mmol/L	3.5-5.1 mmol/L
Chloride (serum)	104 mmol/L	101 mmol/L	98-107 mmol/L
Bicarbonate (serum)	26 mmol/L	26 mmol/L	21-31 mmol/L
Calcium (serum)	9.9 mg/dl	9.2 mg/dl	8.8-10.6 mg/dl
Phosphorous (serum)	3.1 mg/dl	4.8 mg/dl	2.5-4.5 mg/dl
Magnesium (serum)	1.4 mg/dl	1.5 mg/dl	1.9-2.5 mg/dl
Total protein (serum)	6.7 g/dl	6.9 g/dl	6.6-8.3 g/dl
Albumin (serum)	3.2 g/dl	3.2 g/dl	3.5-5.2 g/dl
Globulin (serum)	3.5 g/dl	3.7 g/dl	2.5-3.0 g/dl
Albumin/globulin ratio (serum)	0.9	0.9	1.4-1.7
SGOT (serum)	19 IU/L	24 IU/L	<31 IU/L
SGPT (serum)	18 IU/L	9 IU/L	<34 IU/L
Alkaline phosphatase (serum)	64 IU/L	61 IU/L	30-120 IU/L

**Figure 1:**
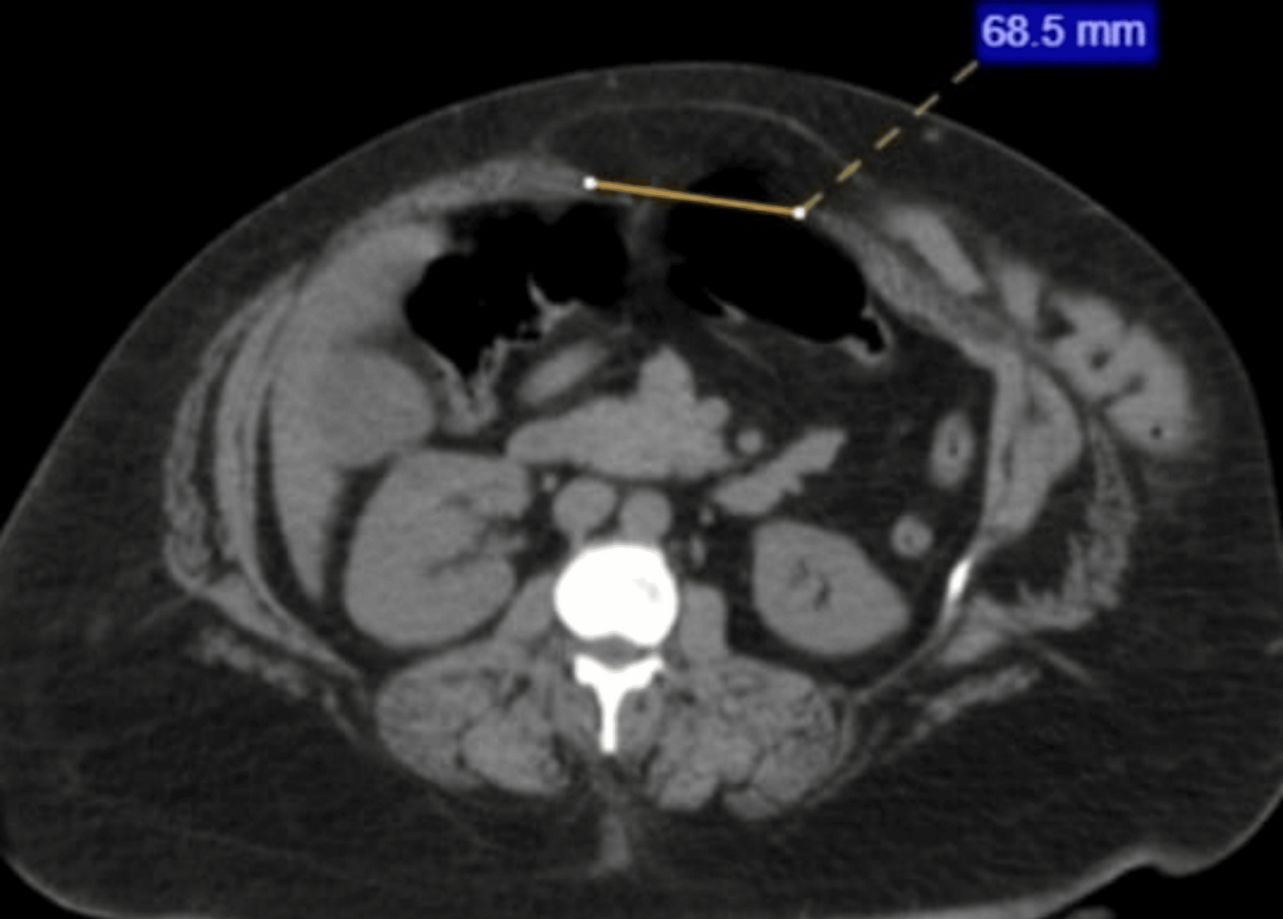
Contrast CT of the abdomen A defect of size 6.8 cm is noted in the midline in the epigastric region with fat and bowel as content. CT: computed tomography

**Figure 2:**
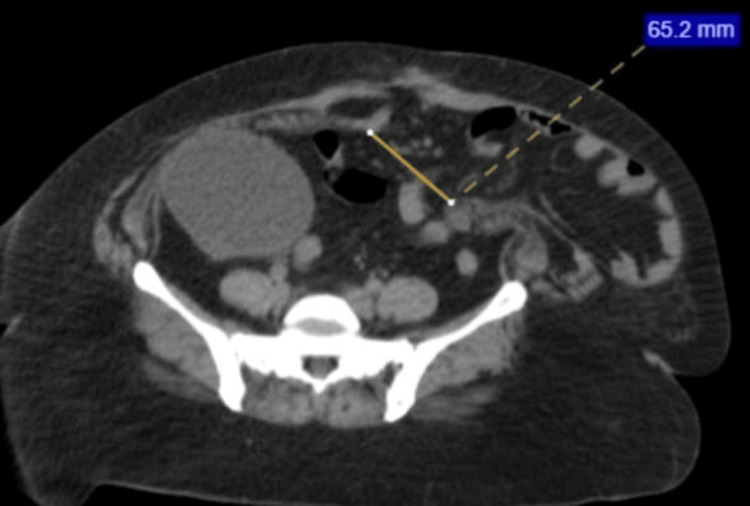
Contrast CT of the abdomen A defect of size 6.5 cm is noted in the midline of the supraumbilical region with fat and bowel as content. CT: computed tomography

**Figure 3:**
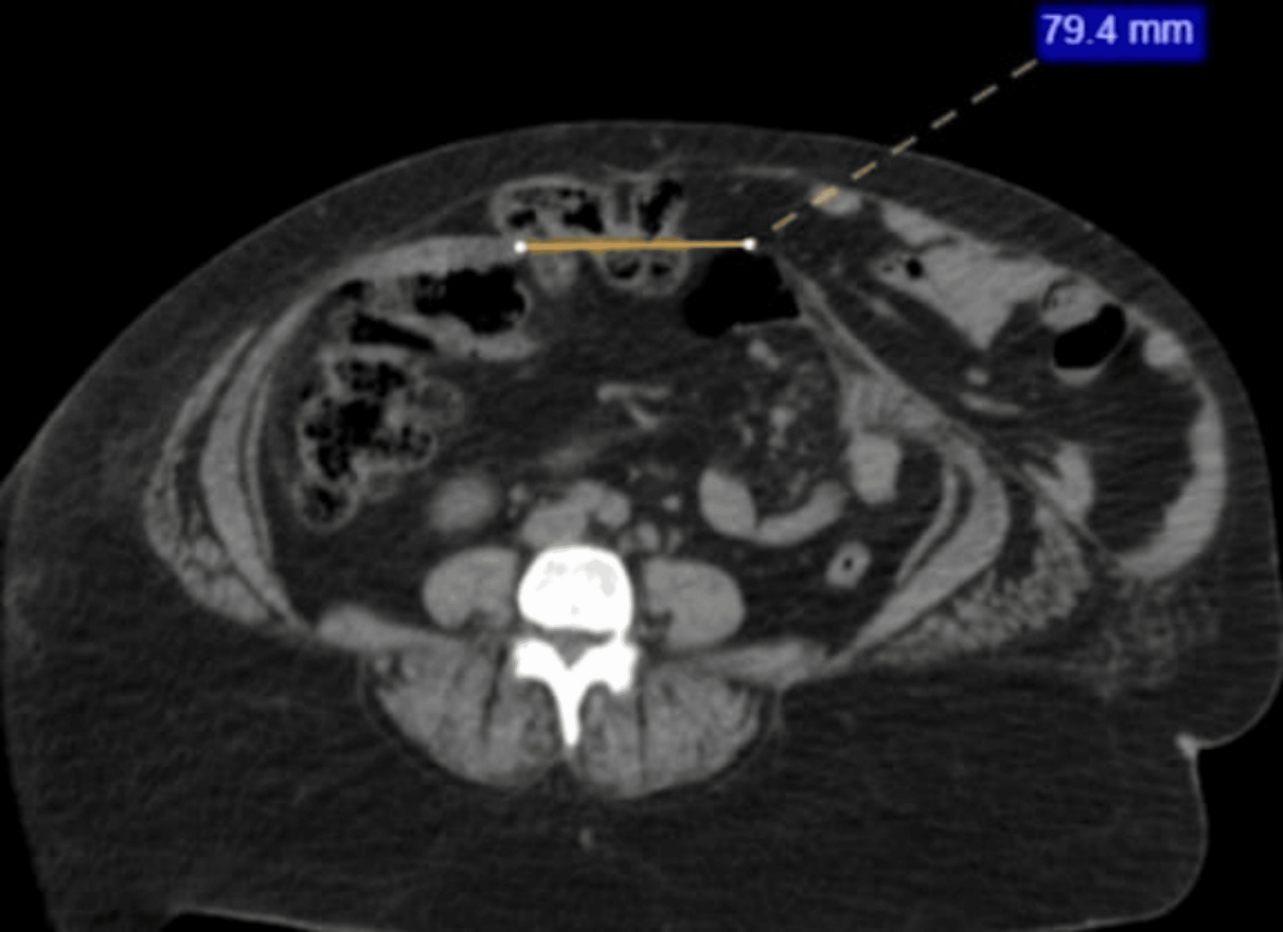
Contrast CT of the abdomen A defect of size 7.9 cm is noted in the midline in the hypogastric region with fat and bowel as content. CT: computed tomography

ECHO and chest X-ray appeared to be normal. Contrast CT of the abdomen revealed multiple anterior abdominal wall defects; it was then diagnosed as an incisional hernia. A defect in the epigastric region (Figure 1), a defect in the supraumbilical region (Figure 2), and a defect in the hypogastric region (Figure 3) were noted.

Several defects were identified in the anterior abdominal wall. The patient requires an emergency surgical procedure to prevent further complications. On the eighth day of hospital admission, exploratory laparotomy with adhesiolysis, rectus mentoplasty, and bilateral transverse abdominis muscle release surgery were done. The intraoperative and postoperative periods were uneventful. The patient was catheterized following the surgery. On postoperative day 4, the patient developed an episode of fever (T-101°C) with desaturation (Spo2 <90% at room air). So the patient was started on oxygen support. When examined locally, the wound was healthy with a 5 to 10 ml discharge (serosanguinous). All routine blood investigations (Table 1) along with new sets of blood cultures were sent. Decreased hemoglobin levels may be attributed to intraoperative blood loss and the postoperative drain, while an increase in white blood cell count indicates the presence of infection. The patient was started on empirical antibiotics, piperacillin/tazobactam and amikacin. Blood culture was done by the automated BACT/ALERT-3D method. It showed a positive signal, and it was cultured on blood agar, chocolate agar, and MacConkey Agar.

Microbiological investigations

**Figure 4:**
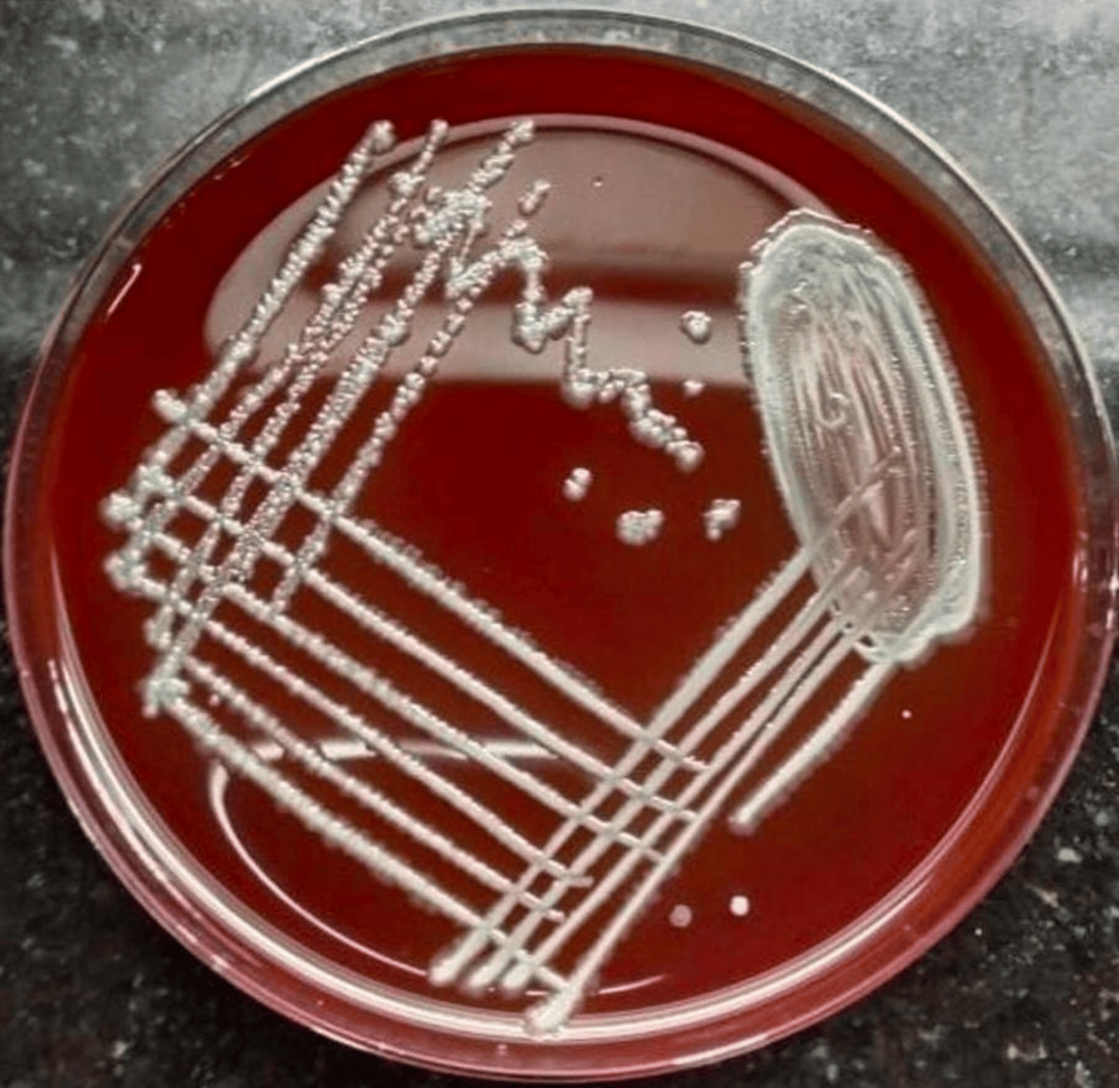
Blood agar with non-hemolytic colonies After 24 hours of incubation, blood agar shows smooth, glistening, non-hemolytic colonies.

The blood agar plate showed non-hemolytic gray colonies (Figure 4).

**Figure 5:**
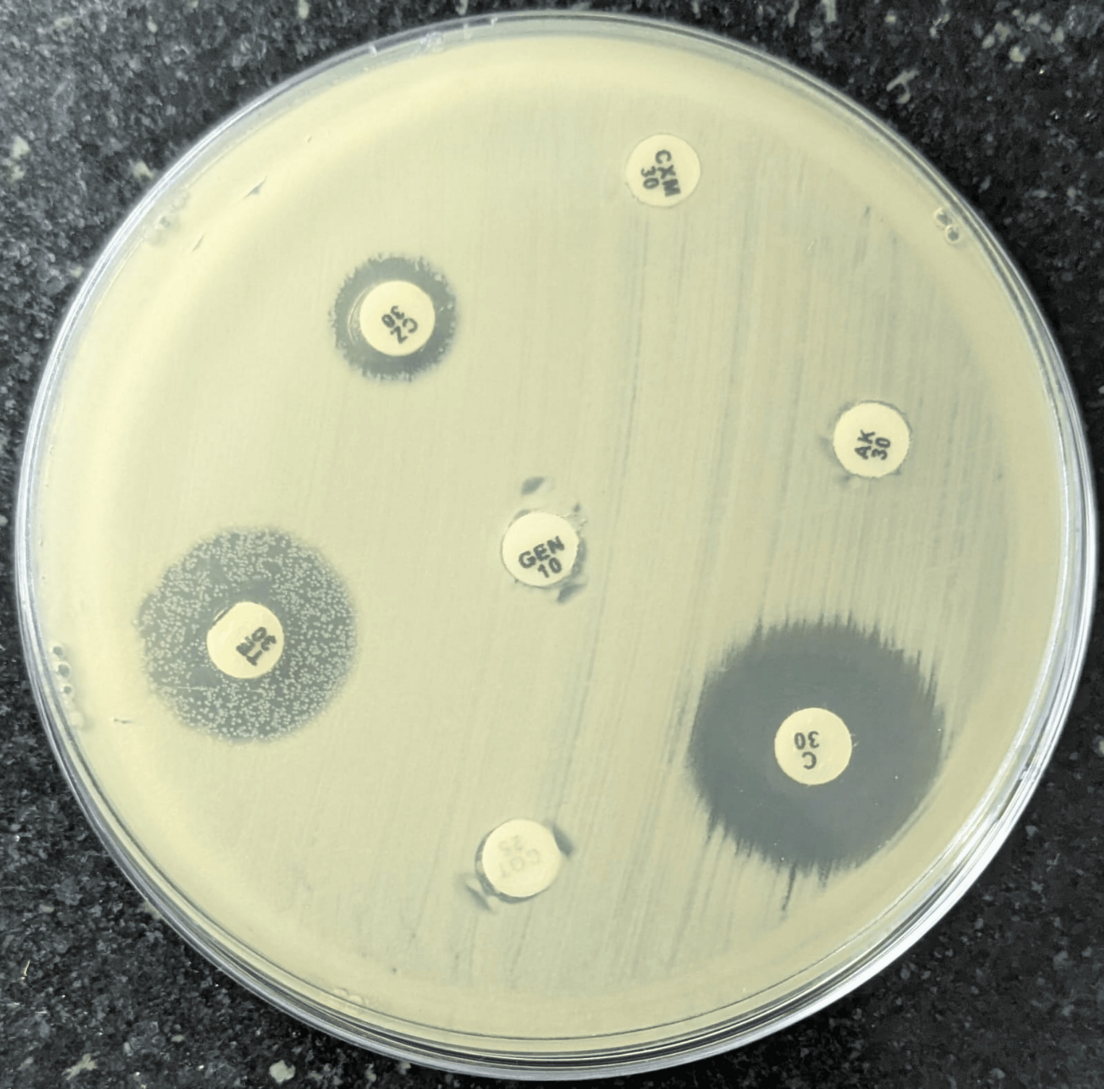
AST using the Kirby-Bauer DD method The zone of inhibition indicates that the organism is susceptible to that antibiotic. The organism showed resistance to gentamicin, amikacin, cefuroxime, cefazolin, tetracycline, and cotrimoxazole, but was susceptible to chloramphenicol. DD values are mentioned in Table 2. AST: antimicrobial susceptibility testing, DD: disk diffusion

Grey colonies were observed on chocolate agar, and pale non-lactose fermenting colonies appeared on MacConkey agar. The organism was gram-negative, non-motile, oxidase, and catalase-positive. Then the isolates were subjected to identification by Vitek 2 Compact System (BIOMÉRIEUX, Marcy-l'Étoile, France) using non-fermenting card code N 406, which was identified as *A. xylosoxidans* with their biochemical properties. It showed citrate and urease negative, the triple sugar iron test showed an alkaline slant, no color change in the butt (K/NC), and the mannitol motility medium showed a non-fermenting, motile organism (NF/M). Antimicrobial susceptibility testing (AST) was done by both the disk diffusion (DD) method and the broth microdilution method. The antibiogram pattern of the identified organism was obtained by the Kirby-Bauer DD method (Figure 5).

The minimum inhibitory concentrations (MICs) of the antibiotics tested were interpreted using the Clinical and Laboratory Standards Institute (CLSI) guidelines for other non-*Enterobacteriaceae* because there are no clear guidelines for sensitivity interpretation of *Achromobacter *isolates (Tables 2-3).

**Table 2:** EUCAST guidelines of 2022 for AST It shows DD zone diameter and MIC breakpoints for piperacillin/tazobactam, meropenem, and trimethoprim-sulfamethoxazole drugs. S: susceptible, R: resistant, EUCAST: European Committee on Antimicrobial Susceptibility Testing, DD: disk diffusion, MIC: minimum inhibitory concentration, AST: antimicrobial susceptibility testing

Antibiotics	DD zone diameter (mm)	MIC breakpoints (mg/L)	Interpretation - EUCAST
Piperacillin/tazobactam	30	4	S
Meropenem	32	1	S
Trimethoprim-sulfamethoxazole	6	>0.125	R

**Table 3:** CLSI guidelines of 2024 for AST It shows the MIC breakpoints. S: susceptible, R: resistant, CLSI: Clinical and Laboratory Standards Institute, MIC: minimum inhibitory concentration, AST: antimicrobial susceptibility testing

Antibiotics	MIC breakpoints (µg/ml)	Interpretation CLSI
Ceftazidime	≤8	S
Piperacillin/tazobactam	≤16/4	S
Cefotaxime	≤8	S
Amoxicillin/clavulanate	≤16/2	S
Cefepime	≤8	S
Ceftriaxone	≤8	S
Imipenem	≤4	S
Meropenem	≤4	S
Ciprofloxacin	≤1	S
Chloramphenicol	≤8	S
Amikacin	≥64	R
Gentamicin	≥16	R
Tetracycline	≥16	R
Trimethoprim-sulfamethoxazole	≤2/38	R

Based on these findings, the patient was treated with intravenous antibiotics piperacillin/tazobactam and meropenem for five days. Following treatment, the repeat blood culture showed no bacterial growth. The patient responded well to the treatment and was discharged with appropriate follow-up instructions. The patient was advised to have a soft, solid diet and to continue with the oral antibiotic amoxicillin/clavulanate for five days and a supportive abdominal binder. Then, the patient was asked to follow up in the general surgery outpatient department after five days.

## Discussion

*A. xylosoxidans*, formerly known as *Alcaligenes xylosoxidans*, is a motile, gram-negative bacillus, a non-fermenter with relatively low pathogenicity. It is primarily found in coastal environments and differs from *Pseudomonas* species by possessing peritrichous flagella. This opportunistic pathogen is uncommon and has modest virulence [10-12]. Although* A. xylosoxidans *is not commonly found in typical human flora, it can thrive in environments such as well water, sterile saline, dialysis fluid, humidifier water, and intravenous fluids. This bacterium is known to survive several disinfectants, including alcohol and chlorhexidine. Due to these characteristics, *A. xylosoxidans* can persist in ICUs and potentially cause nosocomial infection outbreaks, especially when infection control practices are not strictly followed. It is considered an opportunistic pathogen that can cause severe infections in individuals with specific risk factors, such as the presence of an intravenous catheter, cancer, neutropenia, HIV infection, prolonged hospital stays, and in newborns.

The fatality rate for infections caused by* A. xylosoxidans *ranges from 30% in adult sepsis cases to 80% in neonatal sepsis cases [13,14] in a study by Duggan et al.

**Table 4:** Previous publication describing patients with A. xylosoxidans bacteremia In this study, 15 out of 77 patients (19%) had bacteremia related to an intravenous catheter. *A. xylosoxidans*: *Achromobacter xylosoxidans*

Syndrome	No. (%) of patients
Primary bacteremia (no source was identified)	15 (19)
Intravascular catheter-associated bacteremia	15 (19)
Pneumonia	12 (16)
Gastrointestinal or biliary tract infection	5 (6)
Endocarditis	4 (5)
Meningitis	4 (5)
Urinary tract infection	3 4)
Septic arthritis	1 (1)
Soft-tissue infection	1 (1)
Unknown	17 (22)

In this case report, the detection of *A. xylosoxidans* infection in a patient with an incisional hernia was incidental. The patient's risk of developing sepsis was increased due to the use of a Foley catheter and an oxygen face mask post-surgery, compounded by a history of co-morbidities and previous surgery, indicating an already immunocompromised state. The patient had a fever episode only after the surgery (on post-op day 4), which may indicate a healthcare-associated infection, likely due to insufficient infection control practices, mainly improper hand hygiene before and after catheterization, and a lack of catheter care. The patient responded well to piperacillin/tazobactam and meropenem, which were effective due to their strong susceptibility profiles and were considered promising treatments for *A. xylosoxidans*-induced bacteremia.

With resistance to most β-lactam antibiotics and aminoglycosides,* A. xylosoxidan*s displays an unusual sensitivity pattern. Recommended therapeutic options include carbapenems, cotrimoxazole, and antipseudomonal penicillins [15].

In Otta et al., *Achromobacte*r was susceptible to meropenem, trimethoprim-sulfamethoxazole, and piperacillin-tazobactam. The patient showed improvement following treatment with amikacin and piperacillin-tazobactam [16]. In most cases, adequate antimicrobial treatment is essential for managing *A. xylosoxidans* infections. Both *A. xylosoxidans and Achromobacter denitrificans* are clinically significant species often responsible for infections in humans. As an opportunistic pathogen,* A. xylosoxidans *commonly affects individuals with immunosuppression or underlying health conditions. Reported infections include peritonitis, bacteremia, pneumonia, meningitis, abscesses, urinary tract infections, osteomyelitis, corneal ulcers, and prosthetic valve endocarditis [17]. Despite its low pathogenicity, prompt identification and treatment of *A. xylosoxidans* infections are crucial to prevent severe outcomes and fatalities.

## Conclusions

According to the case report finding, this may be the first case of* A. xylosoxidans* bacteremia reported in an incisional hernia patient. It is usually disregarded for its possible pathogenic significance and is thought to be a benign colonizer. Ignoring the bacteria can result in several opportunistic infections in people with impaired immune systems, which can complicate treatment. Therefore, early identification of *A. xylosoxidans* is consequently required to test for sensitivity and determine the optimal course of antibiotic therapy. In order to successfully manage and lower the prevalence of hospital-acquired infections caused by* A. xylosoxidans, *the study emphasizes the significance of comprehensive infection control measures as well as the necessity of ongoing monitoring and adaptation.

Cefiderocol is a new-generation cephalosporin with in vitro activity against carbapenem-resistant gram-negative non-fermenters. Acquired carbapenem resistance occurs via metallo-β-lactamase in *Achromobacter *species, limiting the utility of new β-lactam/β-lactamase inhibitors against carbapenem-resistant* Achromobacter* isolates. A new and emerging antibiotic combination, meropenem-vaborbactam, has been used to treat *Achromobacter* infections in several cases.
